# Pitfalls of NMDA Receptor Modulation by Neuroactive Steroids. The Effect of Positive and Negative Modulation of NMDA Receptors in an Animal Model of Schizophrenia

**DOI:** 10.3390/biom11071026

**Published:** 2021-07-14

**Authors:** Kristina Holubova, Marketa Chvojkova, Barbora Hrcka Krausova, Vojtech Vyklicky, Eva Kudova, Hana Chodounska, Ladislav Vyklicky, Karel Vales

**Affiliations:** 1 National Institute of Mental Health, Topolova 748, 25067 Klecany, Czech Republic; marketa.chvojkova@nudz.cz (M.C.); Karel.Vales@nudz.cz (K.V.); 2 Institute of Physiology CAS, Videnska 1083, 14220 Prague, Czech Republic; barbora.krausova@fgu.cas.cz (B.H.K.); vojtech.vyklicky@fgu.cas.cz (V.V.); Ladislav.Vyklicky@fgu.cas.cz (L.V.); 3 Institute of Organic Chemistry and Biochemistry CAS, Flemingovo namesti 542/2, 16000 Prague, Czech Republic; eva.kudova@uochb.cas.cz (E.K.); hana.chodounska@uochb.cas.cz (H.C.)

**Keywords:** neurosteroids, schizophrenia, MK-801, cognition, anxiety, stress

## Abstract

Evidence from clinical and preclinical studies implicates dysfunction of *N*-methyl-*D*-aspartate receptors (NMDARs) in schizophrenia progression and symptoms. We investigated the antipsychotic effect of two neuroactive steroids in an animal model of schizophrenia induced by systemic application of MK-801. The neuroactive steroids differ in their mechanism of action at NMDARs. MS-249 is positive, while PA-Glu is a negative allosteric NMDAR modulator. We hypothesized that the positive NMDA receptor modulator would attenuate deficits caused by MK-801 co-application more effectively than PA-Glu. The rats were tested in a battery of tests assessing spontaneous locomotion, anxiety and cognition. Contrary to our expectations, PA-Glu exhibited a superior antipsychotic effect to MS-249. The performance of MS-249-treated rats in cognitive tests differed depending on the level of stress the rats were exposed to during test sessions. In particular, with the increasing severity of stress exposure, the performance of animals worsened. Our results demonstrate that enhancement of NMDAR function may result in unspecific behavioral responses. Positive NMDAR modulation can influence other neurobiological processes besides memory formation, such as anxiety and response to stress.

## 1. Introduction

Schizophrenia is a neurodevelopmental and neurodegenerative disorder [1] with symptoms commonly described as positive (hallucinations, delusions), negative (social withdrawal, apathy, amotivation) and cognitive (learning, memory, attention deficits). The genetic, pharmacological and postmortem studies indicate abnormal glutamate transmission and NMDAR hypofunction as a core mechanism underlying schizophrenia onset [2]. The altered function of NMDARs residing on interneurons is believed to cause disinhibition of primary excitatory projections, manifested by schizophrenic symptoms due to distorted excitatory–inhibitory balance [3].

Ionotropic NMDARs are frequently implicated in aberrant brain functioning, likely due to their unique kinetic properties. NMDARs activation requires membrane depolarization necessary for the release of Mg^2+^ block that occurs concurrently with glutamate and glycine binding to the receptor. In addition to Na^+^ and K^+^, NMDARs are also permeable to Ca^2+^ ions [4]. Ca^2+^ ions serve as the intracellular second messenger [5] triggering activation of downstream cascades, mediating synaptic strengthening on one side [6] and caspase activation and apoptosis on the other side [7,8]. Thus, activation of NMDARs has far-reaching consequences beyond membrane depolarization. Furthermore, NMDARs possess a high affinity for glutamate (activated at 0.5 μM concentration) and are likely activated by the ambient extracellular glutamate levels [9,10]. Together with a slower desensitization rate, longer channel-open time and large conductance [11,12], it is not surprising that NMDAR hypofunction or overstimulation can result in brain dysfunction, making these receptors promising therapeutic targets. Current antipsychotic treatment satisfactorily addresses positive schizophrenia symptoms. However, the poor treatment of cognitive symptoms and frequently reported adverse side effects prompted the search for better tolerated and cognition-enhancing treatments.

The research on neurosteroids revealed their wide range of potential clinical applications for the treatment of schizophrenia [13,14,15,16], depression [17], anxiety disorders [18], acute traumatic brain injury [19] or cognitive deficits [20]. As neurosteroids are endogenous steroids locally synthesized in the brain, they are innate modulators of ligand-gated ion channels, capable of rapidly altering neuronal excitability. However, despite structural similarities among neurosteroids modulating NMDAR activity, they elicit strikingly dissimilar functional outcomes. Observed differences might potentially be attributed to their distinct molecular mechanism of action and multiple binding sites at the receptor [21].

Neurosteroid pregnenolone sulfate (PE-S) is a positive allosteric modulator of NMDARs [22,23] with the binding site within the transmembrane domain of the NMDAR [24] that enhances memory and reverses memory impairment induced by NMDAR blockers in rodents [25,26]. PE-S-induced NMDAR potentiation can also be achieved by the facilitation of presynaptic glutamate release [27]. The preclinical and clinical studies revealed the beneficial effect of PE-S on cognitive function in animal models of schizophrenia [28,29,30], schizophrenic patients [16,30] and aged rats [31]. On the other hand, pregnanolone sulfate (PA-S) is a use-dependent and voltage-independent inhibitor with the binding site at the NMDAR ion channel vestibule [32]. Furthermore, PA-S was shown to inhibit NMDARs by reducing open-channel probability, thereby shifting activated receptors to desensitized states [33]. However, caution needs to be applied in interpreting behavioral effects of PA-S and PE-S, since both neurosteroids also bind to other receptors. PE-S acts at a number of ligand-gated ion and voltage-gated channels (GABA_A_, AMPA kainite, glycine receptors, potassium and sodium channels [34,35,36,37,38]). Similarly, pregnanolone sulfate affects all subclasses of ionotropic glutamate receptors [35] and GABA_A_ receptors (GABA_A_Rs) [34].

The availability of sulfated neurosteroids in the brain is limited due to the permanently charged sulfate moiety affecting their ability to cross the blood–brain barrier. In addition, their sensitivity to endogenous steroid sulfatases further contributes to their reduced concentrations in the nervous tissue [39]. Thus, it is desirable to develop compounds with a similar effect but better pharmacokinetic profile. Therefore, a series of PA-S derived neuroactive steroids with varying affinity to NMDARs and neuroprotective effect was synthesized and tested [40]. A structural analogue of PA-S, pregnanolone glutamate (PA-Glu), demonstrated neuroprotective, antipsychotic and antidepressant effects in animal models. Moreover, PA-Glu was surprisingly devoid of psychomimetic side effects typical for other classes of NMDAR inhibitors [41,42,43].

In the present study, we assessed the behavioral effect of a positive NMDAR modulator, pregnanolone-derived (2*E*,4*E*)-4-(20-oxo-5β-pregnan-3-ylidene) but-2-enoic acid (MS-249), either alone or in co-application with MK-801. Application of a selective non-competitive NMDAR antagonist, MK-801, results in NMDAR hypofunction, leading to positive, negative and cognitive schizophrenia symptom manifestations. The efficacy of MS-249 treatment on MK-801-induced schizophrenia-like behavior was compared with the antipsychotic effect of negative NMDAR modulator PA-Glu. Since NMDARs play a key role in neuronal plasticity, learning and memory, their potentiation may result in memory enhancement and antipsychotic effects. We hypothesized that enhancement of NMDAR function by MS-249 would ameliorate deficits induced by NMDAR channel blocker MK-801 more effectively than PA-Glu.

## 2. Materials and Methods

### 2.1. Animals

Male Wistar and Long–Evans rats (350–400 g) used in the experiments were obtained from the Institute of Physiology, an accredited breeding colony originating from Charles River Laboratories, Inc., or purchased from the Velaz Ltd. breeding colony and housed at the National Institute of Mental Health. Animals were housed in transparent plastic cages (20 × 25 × 40 cm) in an air-conditioned animal facility with constant temperature and humidity, and a 12/12 light/dark cycle. Water and food were freely available. Behavioral experiments were pursued during daylight hours. All procedures were performed in accordance with Czech and European legislation regarding the treatment of laboratory animals (Directive 63/2010/UE). Wistar rats (*n* = 355) were employed in all behavioral tests except for the carousel maze, where Long–Evans rats were used instead (*n* = 105). A different set of animals was used for each behavioral test. The number of animals per group is provided in Supplementary Materials (Table S1).

### 2.2. Neuroactive Steroids

Neuroactive steroids used for behavioral assessment were dissolved in β-cyclodextrin (CDX) in 0.01–10 mg/kg concentrations. The neuroactive steroids were administered *i.p.* 30 min prior to the behavioral testing either alone or in combination with MK-801 at the dose of 0.1 or 0.3 mg/kg for pre-pulse inhibition test. CDX was injected 30 min prior to behavioral testing at a volume of 1 mL/kg of body weight as a control. All animals received the same volume of liquid per 1 kg of body weight.

**Figure 1:**
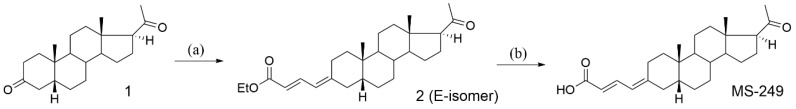
Synthesis of MS-249. Reaction conditions: (**a**) NaH, triethyl 4-phosphonocrotonate, THF, from 0 °C to room temperature; (**b**) KOH, H_2_O, EtOH.

Compound PA-Glu was prepared according to the literature [44]. Compound MS-249 was prepared as described in Figure 1.

All commercial reagents and solvents were used without purification. Melting points were determined with a Hund/Wetzlar micromelting point apparatus (Wetzlar, Germany) and are uncorrected. Proton and carbon NMR spectra were measured in a Bruker AVANCE III™ (Billerica, MA, USA) 400 MHz with chemical shifts given in parts per million (ppm) (δ relative to residual solvent peak for 1H and 13C). Coupling constants (J) are given in Hz. The MS spectra were performed with LCQ Advantage (Thermo Fisher Scientific, Waltham, MA, USA) using ESI mode. Thin-layer chromatography (TLC) was performed on silica gel (Merck, Kenilworth, NJ, USA, 60 µm). For column chromatography, neutral silica gel 60 µm (Fluka) was used. Analytical samples were dried over phosphorus pentoxide at 50 °C/0.25 kPa.

Ethyl (2*E*,4*E*)-4-(20-oxo-5β-pregnan-3-ylidene) but-2-enoate (compound 2). To a suspension of NaH (50% dispersion in paraphine oil; 72 mg, 1.5 mmol) in THF (5 mL) at 0 °C under argon atmosphere, a solution of triethyl 4-phosphonocrotonate (0.39 mL, 1.8 mmol) was added. The reaction mixture was allowed to attain room temperature and then stirred for an additional 30 min. Compound 1 (224 mg, 0.71 mmol, CAS 128-23-4, Carbosynth Ltd., Compton, UK, catalogue number FD21938) in dry THF (5 mL) was added dropwise during 20 min at 0 °C and the mixture was stirred at 0 °C for 2 h. After standing overnight at room temperature under argon atmosphere, the reaction mixture was poured into saturated solution of ammonium chloride (20 mL) and extracted with ethyl acetate (2 × 15 mL). The combined organic extracts were washed with saturated solution of ammonium chloride (10 mL), water (2 × 10 mL) and dried over Na_2_SO_4_. Solvents were evaporated in vacuo. The residue was purified on silica gel using preparative TLC (20% ether in petroleum ether) affording compound 2 (E-isomer, 48 mg, 16%), Z-isomer (63 mg, 22%) and a starting material (13.5 mg, 6%). Compound 2 (E-isomer): m.p. 127–129 °C (petroleum ether/ether); [α]D25 + 124.7 (c 0.26, CHCl_3_). For 1H NMR (400 MHz, CDCl_3_): δ 0.60 (3H, s, H-18), 0.94 (3H, s, H-19), 1.30 (3H, t, J = 7.0 (OCH_2_CH_3_), 2.12 (3H, s, H-21), 2.52 (1H, t, J = 9.0, H-17), 2.61–2.71 (2H, m, H-2β and H-4β), 4.20 (2H, q, J = 7.0, OCH_2_CH_3_), 5.78 (1H, bd, J = 15.2, H-γ, butenoate moiety), 5.94 (1H, dt, J1 = 11.6, J2 = 1.8, H-α, butenoate moiety) and 7.61 (1H, dd, J1 = 15.2, J2 = 11.6 (H-β, butenoate moiety). For 13C NMR (150 MHz, CDCl_3_): δ 209.62 (C-20), 167.83 (COOEt), 155.02 (C-3), 140.29 (C-β, butenoate moiety), 120.24 (C-γ, butenoate moiety), 118.51 (C-α, butenoate moiety), 63.84 (C-17), 60.09 (OCH_2_CH_3_), 56.72 (C-14), 45.47 (C-5), 44.32 (C-13), 40.39 (C-9), 39.20 (C-12), 38.08 (C-4), 38.00 (C-1), 35.70 (C-8), 35.66 (C-10), 31.56 (C-21), 26.84 (C-6), 26.17 (C-7), 24.73 (C-2), 24.41 (C-15), 23.24 (C-19), 22.82 (C-16), 20.99 (C-11), 14.34 (OCH_2_CH_3_) and 13.43 (C-18). IR spectrum (CHCl_3_): 3060, 981 (=CH); 1699 (C = O, COOH and ketone); 1633, 1613 (C = C); and 1150, 1043 (CH_3_, OCH_2_CH_3_). MS: EI m/z 435 (75%, M + Na), 413 (23%, M + 1) and 367 (38%, M—OCH_2_CH_3_). For C_27_H_40_O_3_ (412.6) calculated: 78.60% C, 9.77% H; found: 78.58% C, 9.82% H.

(2E,4E)-4-(20-Oxo-5β-pregnan-3-ylidene) but-2-enoic acid (MS-249). To a solution of compound 2 (67 mg 0.15 mmol) in dry benzene (3 mL), ethylene glycol (0.11 mL, 1.97 mmol), triethyl orthoformate (0.135 mL, 1.27 mmol) and p-toluenesulfonic acid mono-hydrate (1 mg, 0.006 mmol) were added at room temperature and the reaction mixture was stirred for 12 h. Then, it was poured into ice water and extracted with ethyl acetate (60 mL), washed with aqueous solution of NaHCO_3_, water, dried and evaporated. The crude product was purified by preparative TLC (petroleum ether/ether (4:1) and two drops of pyridine). The isolated product (0.09 mmol) in methanol (5 mL) was hydrolyzed in a solution of potassium hydroxide (1.87 mmol), water (0.24 mL) and ethanol (0.24 mL) under the reflux. The reaction mixture was poured into water with crushed ice and acidified by a mixture HCl/H_2_O (1:2) to pH 1, then the precipitate was filtered off, washed with water (2×) and dried overnight at 40 °C. The solid was crystallized from a mixture of acetone–water affording compound MS-249 (34 mg, 55%): m.p. 170–172 °C, [α]D25 +123.7 (c 0.24, CHCl_3_). For 1H NMR (400 MHz, CDCl_3_): 0.62 s, (3H, s, H-18), 0.95 (3H, s, H-19), 2.12 (3H, s, H-21), 2.55 (1H, t, J = 9, H-17), 2.64–2.76 (2H, m, H-2β and H-2β), 5.79 (1H, d, J = 15.1, H-γ, butenoate moiety), 5.98 (1H, d, J = 11.7, H-α, butenoate moiety) and 7.7 (1H, dd, dd, J1 = 15, J2 = 11.7, H-β, butenoate moiety). IR spectrum (CHCl3): 3527 (OH, COOH, monomer); 3090 (OH, COOH, dimer); 1695 (C = O, ketone); 1686 (C = O, COOH, dimer); and 1631, 1610 (C = C) 1285 (C-O, COOH, dimer). MS: EI m/z 384 (53%, M), 366 (16%, M—H_2_O), 341 (8%, M—CH_3_CO), 323 (10%, M—COOH, O), 299 (14%, M—CH = CH = CH-COOH, H) and 257 (7%, M—CH = CH = CH-COOH, CH_3_CO). For C_25_H_36_O_3_ (384.5) calculated: 78.08% C, 9.44% H; found: 77.96% C; 9.61% H.

### 2.3. Electrophysiology

Electrophysiological experiments were performed on human embryonic kidney (HEK293) cells transfected with cDNAs encoding GluN1-1a/GluN2B/GFP genes as de-scribed previously [40]. Whole-cell voltage-clamp recordings were measured at a holding potential of −60 mV, with compensation of capacitance and series resistance (<10 MΩ) by 80–90%. Data were obtained using an Axopatch 200B amplifier (Molecular Devices, Sunnyvale, CA, USA), sampled at 10 kHz and then filtered at 2 kHz. Pipettes used for patching (3–5 MΩ) were filled with an intracellular solution containing (in mM): 15 CsCl, 120 gluconic acid, 10 BAPTA, 3 MgCl_2_, 1 CaCl_2_, 10 HEPES and 2 ATP-Mg salt (pH was adjusted to 7.2 with CsOH). Extracellular solution (ECS) applied to the HEK293 cells contained the following (in mM): 2.5 KCl, 160 NaCl, 10 HEPES, 0.2 EDTA, 10 glucose and 0.7 CaCl_2_ (pH was adjusted to 7.3 with NaOH). Glycine (10 µM) as co-agonist of NMDARs was present in all control and test solutions. For drug applications was used a multibarrel fast perfusion system controlled by a microprocessor with the rate of solution exchange around the cells of ~10 ms. NMDAR responses were induced by 1 µM or 1 mM glutamate (in case of potentiation or inhibition, respectively). Experiments were performed at room temperature.

MK-801 and steroids were dissolved in dimethyl sulfoxide (DMSO) and added to the ECS at the concentrations indicated in the figures, and the final DMSO concentration in all test and control solutions was adjusted to 1%. An equivalent amount of DMSO was present in control solutions. All used drugs were purchased from Sigma-Aldrich (St. Louis, MO, USA).

The degree of inhibition/potentiation (E) induced by the steroid was determined using the following formula: *E = ((Is − Ia)/Ia)* × 100, where *Is* is the current amplitude during the steroid and glutamate co-application and *Ia* is the current amplitude during the glutamate application. All data are presented as the mean ± standard error of mean (SEM) with *n* corresponding to the number of independent measurements. The statistical comparison was performed using the Student’s *t*-test; the value of *p* < 0.05 was considered significant.

### 2.4. [^35^S]-Tert-Butylbicyclophosphorothionate ([^35^S]TBPS) Displacement

Compounds PA-Glu and MS-249 were tested for their ability to noncompetitively displace [^35^S]TBPS from the picrotoxin-binding site on GABA_A_Rs in the rat cerebral cortex. Membrane homogenates (120 μg protein) were incubated with [^35^S]TBPS (3 nM) with or without PA-Glu/MS-249 at 22 °C for 2 h using a buffer solution as follows: Na_2_HPO_4_/KH_2_PO_4_ (50 mM, pH 7.4), NaCl (500 mM). Nonspecific binding was deter-mined by picrotoxinin (20 μM). After the incubation, samples were filtered through glass fiber filters (GF/B, Packard) presoaked with 0.3% PEI and washed with Tris-HCl (50 mM) using a 96-sample cell harvester (Unifilter, Packard, Palo Alto, CA, USA). The filters were dried and counted in a scintillation counter (Topcount, Packard) using a scintillation cock-tail (Microscint 0, Packard). The filters were dried and then counted for radioactivity in a scintillation counter (Topcount, Packard) using a scintillation cocktail (Microscint 0, Packard). The results are expressed as a percentage of inhibition of the control radioligand-specific binding.

### 2.5. Behavioral Tests

#### 2.5.1. Open Field (OF)

Rats were individually placed in a dimly illuminated open field (50 × 50 cm) for 10 min. The spontaneous locomotor activity was detected by evenly spaced infrared light beams and evaluated as distance walked. Beam interruptions caused by moving animal were registered by software (Multi Conditioning System, TSE Systems, Bad Homburg, Germany).

#### 2.5.2. Elevated Plus Maze (EPM)

The setup for this experiment can be described as two open arms, perpendicularly intersected by two closed arms at the central platform, which is elevated 50 cm above the ground. Both types of arms measure 50 × 10 cm, with the closed arms enclosed by 40 cm tall walls. The rat is initially positioned at the closed arm’s central platform. A camera placed above the apparatus then proceeds to record the distance travelled, time spent in both arms and on the central platform in 5 min intervals. A specialized software was used to analyze acquired data (Noldus, Ethovision, Wageningen, The Netherlands).

#### 2.5.3. Pre-Pulse Inhibition of Acoustic Startle Response (PPI)

PPI was evaluated in sound-proof, evenly lit and ventilated startle chambers (SR-LAB, San Diego Instruments, CA, USA) with a Plexiglas stabilimeter (8.7 cm inner diameter). On the first day, rats were habituated to the startle chamber during a 5 min pre-training session consisting of 5 pulses-only stimuli (115 dB/20 ms) presented over background white noise (75 dB). The testing session took place 2 days after habituation to the chambers. The test session comprised of 72 trials with an inter-trial interval (ITI) of 4–20 s (mean ITI: 12.27 s). After 5 min of habituation, when rats were exposed to a continuous 75 dB background white noise, six 125 dB/40 ms pulse-alone trials were delivered to establish baseline acoustic startle response (ASR). Following this, 60 trials were presented in a pseudorandom order: (A) pulse alone: 40 ms/125 dB; (B) pre-pulse–pulse: 20 ms/83 dB or 20 ms/91 dB pre-pulse followed by 40 ms/125 dB pulse with a variable (30, 60, or 120 ms) inter-stimulus interval (ISI: mean = 70 ms); and (C) 60 ms of no stimulus. Finally, six pulse-alone trials were delivered again (125 dB/40 ms). PPI was calculated as follows: [100 − (mean pre-pulse–pulse trials/ mean pulse alone trials) × 100]. Animals with a mean ASR response lower than 10 were excluded from analyses as non-responders. This experiment was carried out at the NIMH on animals purchased from Velaz Ltd. due to the availability of PPI apparatus at this institute.

#### 2.5.4. Step-Through Passive Avoidance (PA)

The arena (50 × 50 cm) consisted of two equally sized compartments divided by a sliding door (Multi Conditioning System, TSE Systems, Bad Homburg, Germany). One compartment was intensively illuminated while the other remained dark. We initially positioned the rat in the illuminated section, from where it was free to move between the two compartments for 5 min, not being administered any shocks at this stage (“the habituation session”). Thirty minutes following the habituation, we placed the rats into the arena again for a “training” period. The training consisted of footshock (2.5 mA, 8 s) administration to the animal via the stainless-steel grid floor once it crossed to the dark compartment, without the possibility to return to the illuminated area. One hour later, a test was conducted to observe the rat’s latency to cross into the dark section during a 5 min, footshock-free period. This test served as an assessment of memory function, followed by an extensive software evaluation.

#### 2.5.5. Carousel Maze (CM)

The cognitive coordination and spatial navigation were assessed in the carousel maze. For a detailed description of the apparatus used, please refer to [45]. Briefly, the metallic circular arena (82 cm in diameter) enclosed by transparent Plexiglass walls (40 cm high) was rotating with a constant speed of 1 round per minute. The invisible 60° to-be-avoided shock sector was defined by the coordinate system of the room. Successful solving of the task required active avoidance of the shock sector, where rats received mild electric footshock upon entering. Rats had to discriminate between relevant distal cues located in the room and irrelevant arena-bound cues. The camera attached to the ceiling above the arena recorded the rat’s movements, and tracking software (Tracker, Bio-Signal Group Corp., Brooklyn, NY, USA) analyzed cognitive parameters (number of entrances into the shock sector and number of shocks rat receives) and distance actively walked by the rat after filtering out arena movements. Rats underwent four 20 min sessions in four consecutive days. Only the fourth day was analyzed because, by this time, the animals had reached an asymptotic level of performance. In this test, the Long–Evans strain was used due to better eyesight and cognitive performance.

#### 2.5.6. Statistical Analysis

Statistical analyses were performed by the statistical software GraphPad Prism 5.0 (San Diego, CA, USA). The treatment effect was determined using one-way ANOVA followed by Dunnett’s post hoc test when appropriate. The selected pairs of experimental groups were compared in the post hoc test—neurosteroid-treated groups were compared to vehicle and MK-801 groups. If the normality assumption was violated, data were transformed to attain Gaussian distribution. However, in the graph, the original values were plotted. Data are presented as the group means ± SEM. The significance level was set at *p* < 0.05.

## 3. Results

### 3.1. Electrophysiology

**Figure 2:**
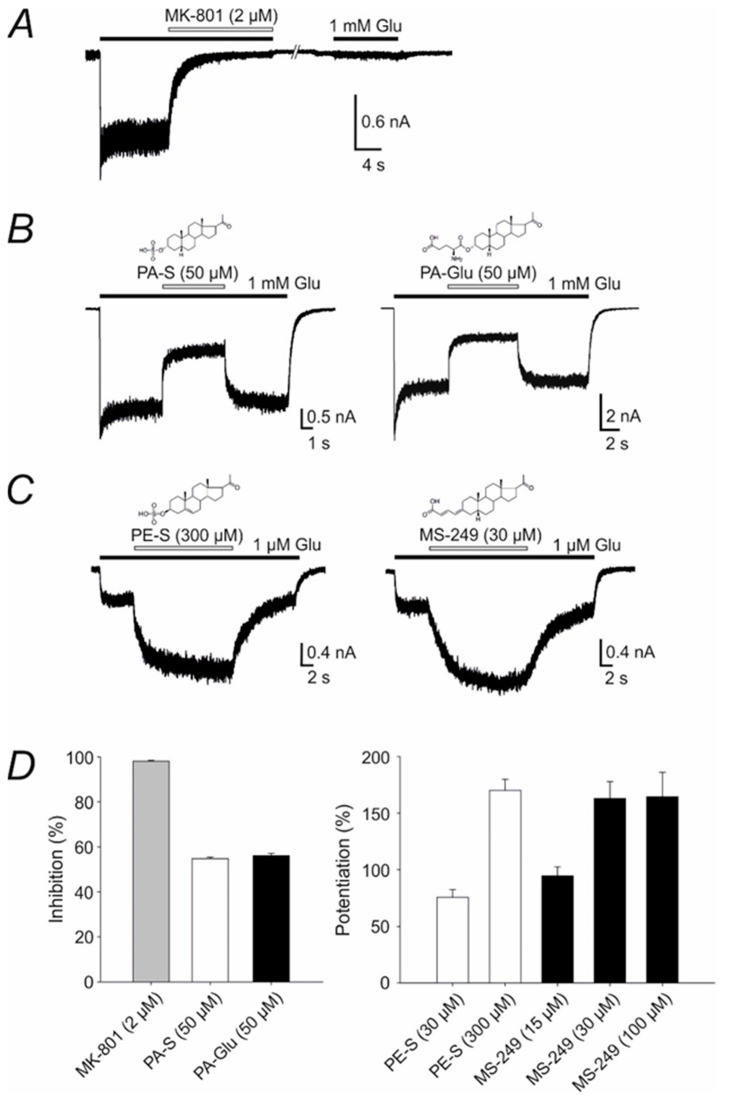
Effect of MK-801 and neuroactive steroids on NMDAR responses. (**A**–**C**) Examples of traces obtained from HEK293 cells expressing GluN1/GluN2B receptors. MK-801, as well as endogenous neurosteroids (PA-S and PE-S) and their synthetic analogues (PA-Glu and MS-249), were applied simultaneously with glutamate (duration of glutamate and compound application is indicated by filled and open bars, respectively). (**D**) Graphs of the mean ± SEM (*n* ≥ 5) of the relative compound effect on the NMDAR responses. Note that MK-801, PA-S and PA-Glu inhibited the NMDAR responses while PE-S and MS-249 potentiated the NMDAR responses.

In accordance with previous electrophysiological experiments, we demonstrated that MK-801 irreversibly inhibits the responses of NMDARs (Figure 2A,D). In agreement with previous in vitro studies, our electrophysiological measurements show that the endogenous neurosteroid PA-S had an inhibitory effect at recombinant NMDARs (Figure 2B). Furthermore, the inhibitory effect of 50 µM PA-Glu at recombinant NMDARs was comparable to that of 50 µM PA-S (Figure 2B,D; *p* = 0.347, Student’s *t*-test).

Following the findings of previous studies, we observed that endogenous PE-S had a potentiation effect at recombinant NMDARs (Figure 2C). Another PA-S analogue, MS-249, potentiated the NMDAR responses (Figure 2C). The potentiation effect of 30 µM MS-249 at NMDARs was comparable to that of 300 µM PE-S (Figure 2C,D; *p* = 0.726, Student’s *t*-test). Unfortunately, we could not perform the dose–response analysis for the MS-249 effect at NMDARs due to its limited solubility (we observed precipitation at 100 µM).

### 3.2. [^35^S]-Tert-Butylbicyclophosphorothionate ([^35^S]TBPS) Displacement

The ability of compounds PA-Glu and MS-249 to modulate GABA_A_ receptor activity was evaluated in the [^35^S]-tert-butylbicyclophosphorothionate ([^35^S]TBPS)-binding assay. The ability of neurosteroids to inhibit [^35^S]TBPS binding has been commonly used to identify neurosteroids’ positive allosteric modulatory activity [46]. For example, the neurosteroid allopregnanolone acts at low nanomolar concentrations as an endogenous modulator of GABA action at GABA_A_Rs [47]. Compounds PA-Glu and MS-249 were first screened at concentrations of 100 nM and 1 µM. Compound MS-249 was not able to demonstrate displacement of [^35^S]TBPS in any of the tested concentrations. Therefore, this compound can be considered inactive at GABA_A_Rs. In contrast, PA-Glu was able to displace [^35^S]TBPS by 8% at concentration 100 nM and by 37% at concentration 1 µM. Therefore, an additional 7-point concentration–response curve was measured (10 nM–100 µM) affording the IC_50_ value for [^35^S]TBPS inhibition of 7.5 µM.

### 3.3. Behavioral Tests

The table summary of results from all behavioral tests is provided in the Supplementary Material (Table S1).

#### 3.3.1. Open Field (OF)

**Figure 3:**
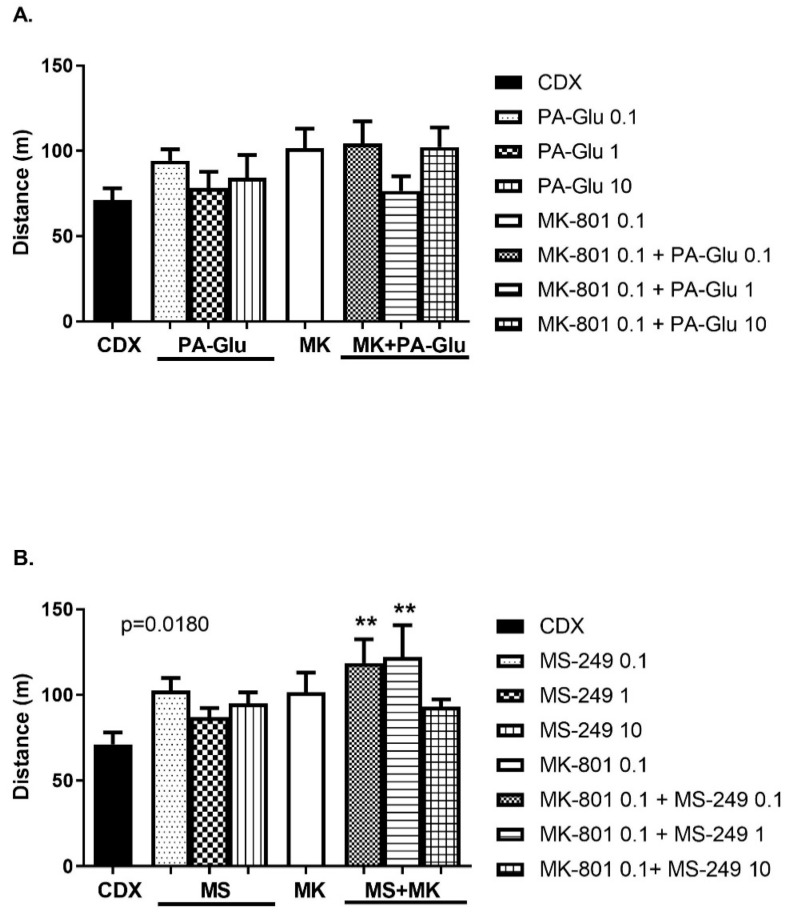
Spontaneous locomotion in OF. Total distance travelled by animals in OF during 10 min analyzed by 1-way ANOVA followed by Dunnett’s post hoc test. (**A**) No effect on performance was detected when PA-Glu was applied alone or in combination with MK-801. (**B**) While MS-249 application alone had no impact on locomotion, MS-249 potentiated the effect of MK-801, resulting in hyperlocomotion compared to controls. ** *p* < 0.01 vs. CDX controls.

One-way ANOVA revealed no significant effect of PA-Glu treatment (Figure 3A) and a significant effect of MS-249 treatment (F(7, 46) = 2.747; *p* = 0.0180) (Figure 3B) on spontaneous activity in the open field. Neither drug applied alone significantly influenced locomotion compared to vehicle-treated rats, as the post hoc test showed. MK-801 0.1 mg/kg administration insignificantly increased locomotion. However, administration of MS-249 enhanced locomotor activity in MK-801-treated rats (Figure 3B). MS-249 potentiated the MK-801 effect, while MK-801 + PA-Glu administration did not further increase locomotion. The post hoc test showed a significant increase in activity in MK-801 + MS-249 0.1 mg/kg (*p* < 0.01) and MK-801 + MS-249 1 mg/kg (*p* < 0.01) treated rats in comparison to controls (Figure 3B).

#### 3.3.2. Elevated Plus Maze (EPM)

**Figure 4:**
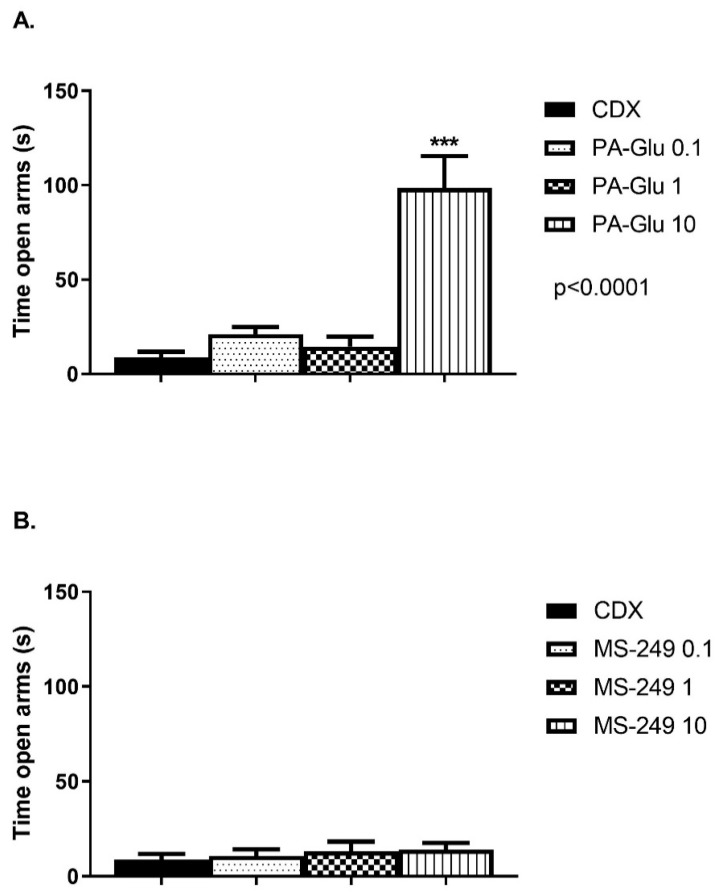
Time spent in open arms in EPM. The time spent in open arms was analyzed as a measure of decreased anxiety. (**A**) PA-Glu administration at the highest dose exerted an anxiolytic effect reflected by a significant increase in time spent in open arms. (**B**) MS-249 did not affect anxiety in EPM. Data were analyzed by 1-way ANOVA followed by Dunnett’s post hoc test. *** *p* < 0.001 vs. CDX controls.

No effect of MS-249 on any parameters analyzed in EPM was detected (Figure 4B, Figure S1B and S2B). PA-Glu administration significantly affected time spent in open arms F(3, 31) = 24.35 (Figure 4A), *p* < 0.0001, latency to enter open arms F(3, 31) = 6.391 (Figure S1A), *p* = 0.0017 and total distance travelled F(3, 31) = 22.43, *p* < 0.0001 (Figure S2A) in EPM. Treatment with 10 mg/kg of PA-Glu significantly increased time spent in open arms (*p* < 0.001), decreased latency to enter open arms (*p* < 0.01) and increased locomotion (*p* < 0.001) in comparison to controls. The latency was also significantly reduced in 0.1 (*p* < 0.01) and 1 mg/kg (*p* < 0.05) PA-Glu treated rats when compared to vehicle-treated rats (Figure S1A).

#### 3.3.3. Pre-Pulse Inhibition of Startle Response (PPI)

**Figure 5:**
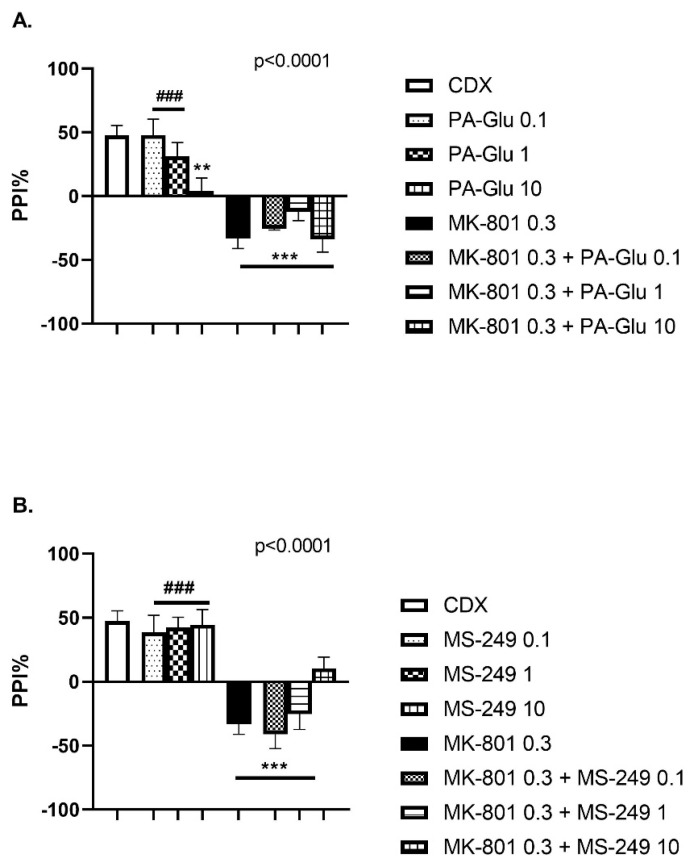
PPI of the acoustic startle response. (**A**) PA-Glu dose-dependently decreased PPI, with 10 mg/kg dose reaching the significance level compared to vehicle-treated rats. PA-Glu failed to reverse MK-801-induced PPI deficits. (**B**) Neither dose of MS caused PPI deficits when applied alone. Co-application of MS with MK-801 did not rescue the performance in PPI. Data were analyzed by 1-way ANOVA followed by Dunnett’s post hoc test. ** *p* < 0.01, *** *p* < 0.001 vs. CDX controls, ^###^ <0.001 vs. MK-801 treated rats.

One-way ANOVA detected a significant effect of PA-Glu (F(7, 50) = 12.63, *p* < 0.0001) (Figure 5A) and MS-249 (F(7, 44) = 12.00, *p* < 0.0001) (Figure 5B) treatment on sensorimotor gating in PPI. The highest dose of PA-Glu worsened the performance when compared to controls (*p* < 0.01). Contrary to PA-Glu, MS-249 had no negative impact on performance when applied alone in either dose. In agreement with the literature, MK-801 application caused PPI deficits (*p* < 0.001 vs. controls) that failed to be reversed by either neuroactive steroid treatment (*p* < 0.001 MK-801 + PA-Glu of all doses vs. controls, *p* < 0.001 MK-801 + MS-249 0.1 and 1 mg/kg vs. controls). In addition, the post hoc test revealed a significant difference between the MK-801 group and PA-Glu 0.1 mg/kg (*p* < 0.001), PA-Glu 1 mg/kg (*p* < 0.001) and MS-249 0.1, 1, 10 mg/kg (all comparisons *p* < 0.001).

#### 3.3.4. Step-Through Passive Avoidance (PA)

**Figure 6:**
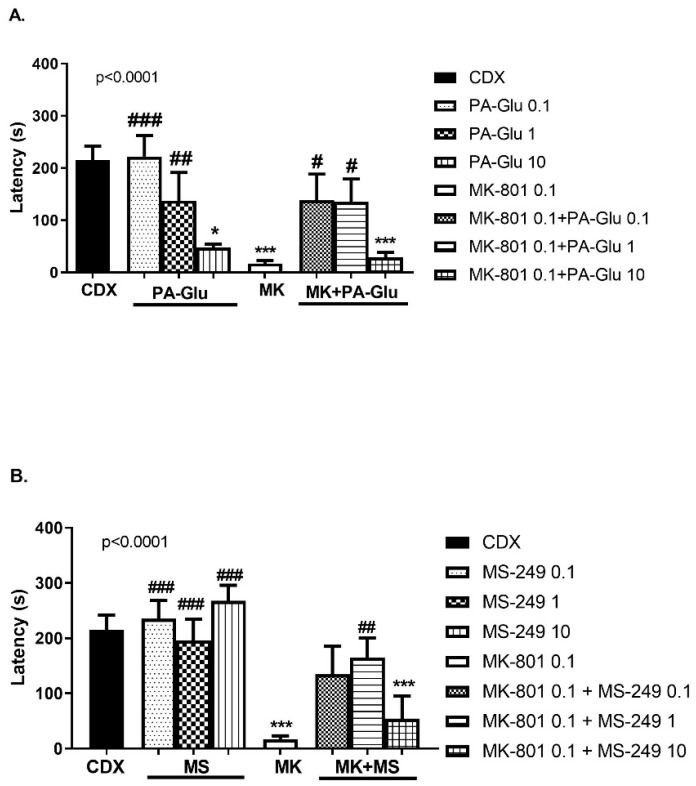
Step-through PA. (**A**) PA-Glu administration dose-dependently decreased latency to enter dark compartment with the highest dose reaching the level of significance when compared to controls. PA-Glu 0.1 and 1 mg/kg reversed cognitive impairment induced by MK-801 co-application. (**B**) Administration of MS-249 of all doses did not negatively affect the latency. MS-249 1 mg/kg dose reversed the memory deficits in MK-801-treated rats. Data were analyzed by 1-way ANOVA followed by Dunnett’s post hoc test. * *p* <0.05, *** *p* < 0.001 vs. CDX controls, ^#^ <0.05, ^##^ <0.01, ^###^ <0.001 vs. MK-801-treated rats.

The administration of PA-Glu (F(7, 51) = 6.622, *p* < 0.0001) (Figure 6A) and MS-249 (F(7, 63) = 9.567, *p* < 0.0001) (Figure 6B) significantly affected the performance in the passive avoidance task. MK-801 administration caused memory impairment reflected by shortened latency to cross into the dark compartment compared to CDX-treated animals (*p* < 0.001). PA-Glu administration dose-dependently decreased latency to enter the dark compartment with the dose of 10 mg/kg reaching significance (*p* < 0.05) compared to controls. The post hoc test revealed a significant difference in latency between MK-801-treated rats and PA-Glu 0.1 (*p* < 0.001) and 1 mg/kg (*p* < 0.05)-treated rats. Treatment with PA-Glu at the doses of 0.1 (*p* < 0.05) and 1 mg/kg (*p* < 0.05) reversed the cognitive deficit induced by MK-801 co-application (Figure 6A). MK-801 + PA-Glu 10 mg/kg co-application failed to rescue the deficit, and the rats’ performance differed from that of controls (*p* < 0.001).

Performance of animals treated with all doses of MS-249 (0.1–10 mg/kg) was not affected; latency was comparable to controls, while there was a significant difference in comparison to MK-801-treated animals (*p* < 0.001 for all doses). MS-249 1 mg/kg reversed the cognitive deficit induced by MK-801 co-application (*p* < 0.01) (Figure 6B). MK-801 and MK-801 + MS-249 10 mg/kg treatment resulted in a worse performance compared to controls (*p* < 0.001).

#### 3.3.5. Carousel Maze (CM)

**Figure 7:**
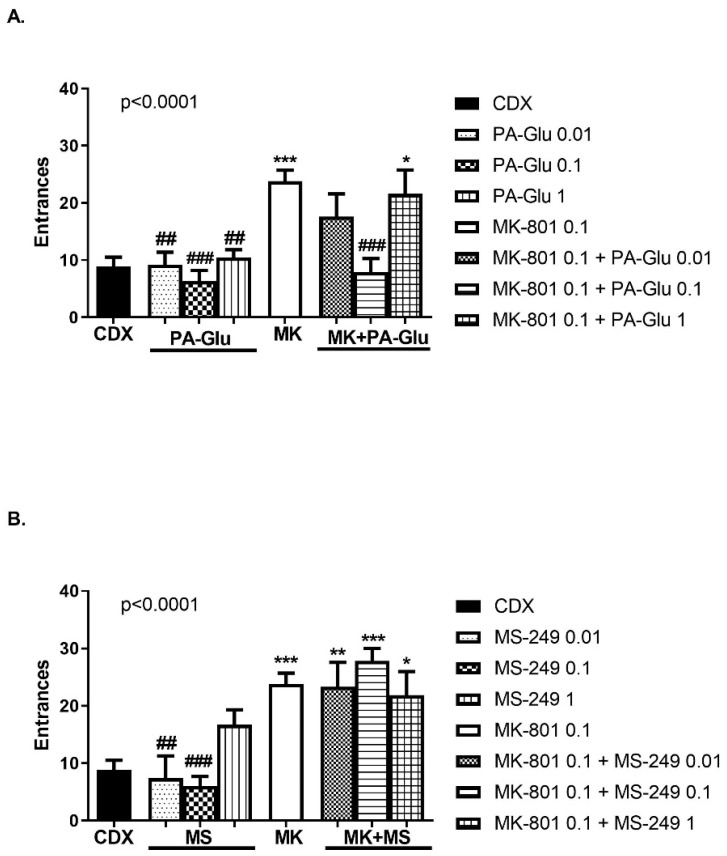
Number of entrances into the shock sector in CM. (**A**) PA-Glu administration alone had no adverse effect on performance. PA-Glu 0.1 mg/kg reversed cognitive deficit induced by MK-801 co-application. (**B**) MS-249-treated groups did not differ significantly from controls. None of the doses applied rescued the performance in MK-801-treated rats. Data were analyzed by 1-way ANOVA followed by Dunnett’s post hoc test. * *p* < 0.05, ** *p* < 0.01, *** *p* < 0.001 vs. CDX controls, ^##^ <0.01, ^###^ <0.001 vs. MK-801-treated rats.

One-way ANOVA detected a significant effect of treatment on cognitive performance in a carousel maze measured by the number of entrances, F(7, 59) = 6.533, *p* < 0.0001 and F(7, 54) = 8.158, *p* < 0.0001 for PA-Glu (Figure 7A) and MS-249 analysis (Figure 7B). MK-801 application resulted in an increased number of entrances (errors) compared to CDX-treated (*p* < 0.001), PA-Glu-treated (*p* < 0.01 vs. PA-Glu 0.01 and 1 mg/kg, *p* < 0.001 vs. PA-Glu 0.1 mg/kg) and MS-249-treated (*p* < 0.01 vs. 0.01 mg/kg and *p* < 0.001 vs. 0.1 mg/kg) animals. PA-Glu 0.1 mg/kg exerted a therapeutic effect in MK-801-induced psychosis by improving cognitive deficit (Figure 7A). The performance of MK-801 + PA-Glu 1 mg/kg-treated rats was significantly worse than that of controls (*p* < 0.05). None of the MS-249 doses attenuated cognitive deficit induced by MK-801 application (*p* < 0.05 CDX vs. MK-801 + MS-249 1 mg/kg, *p* < 0.01 CDX vs. MK-801 + MS-249 0.01 mg/kg and *p* < 0.001 CDX vs. MK-801 + MS-249 0.1 mg/kg). A single administration of either neuroactive steroid did not alter the performance.

**Figure 8:**
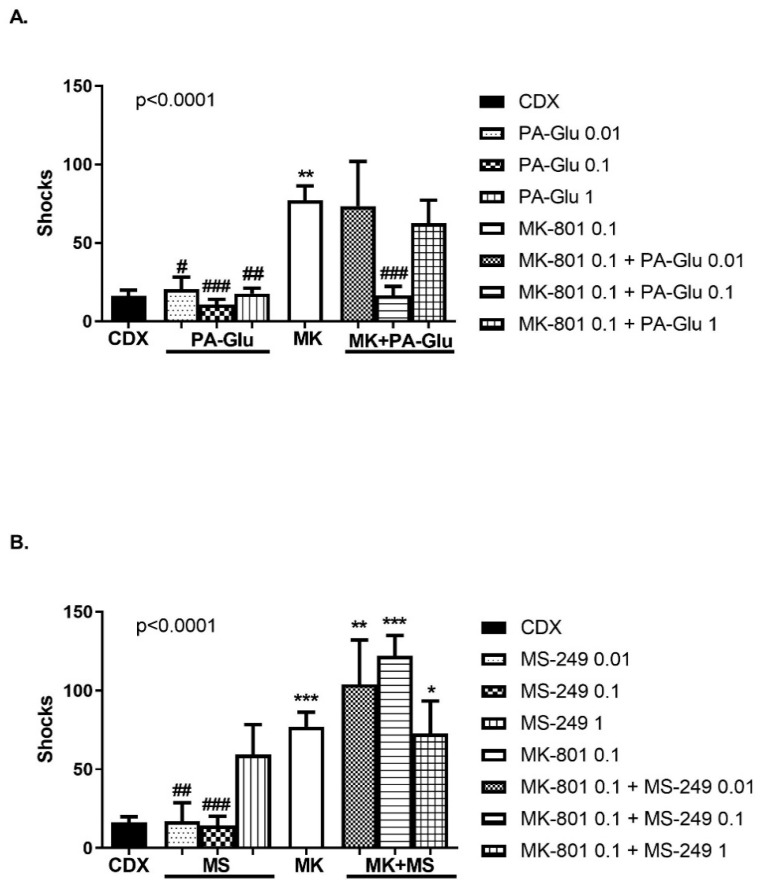
Number of shocks in CM. (**A**) Number of shocks between controls and PA-Glu-treated groups did not differ. PA-Glu 0.1 mg/kg administration reduced the number of shocks after MK-801 co-application to the level of controls. (**B**) The number of shocks in MS-249-treated rats when applied alone was comparable to controls. All MS-249 doses significantly increased the number of shocks when co-applied with MK-801 compared to the control group. Data were analyzed by 1-way ANOVA followed by Dunnett’s post hoc test. * *p* < 0.05, ** *p* < 0.01, *** *p* < 0.001 vs. CDX controls, ^#^ <0.05, ^##^ <0.01, ^###^ <0.001 vs. MK-801-treated rats.

In addition to the number of entrances, we analyzed the number of shocks as well. Neuroactive steroid administration significantly affected the number of shocks received, F(7, 59) = 6.673, *p* < 0.0001 for PA-Glu (Figure 8A) and F(7, 54) = 9.248, *p* < 0.0001 for MS-249 analysis (Figure 8B). Application of MK-801 led to an increased number of shocks when compared to CDX (*p* < 0.01), PA-Glu 0.01 mg/kg (*p* < 0.05), PA-Glu 0.1 mg/kg (*p* < 0.001), PA-Glu 1 mg/kg (*p* < 0.01), MS-249 0.01 (*p* < 0.01) and 0.1 mg/kg (*p* < 0.001). Likewise, in the number of errors, MK-801 + PA-Glu 0.1 administration had a beneficial effect on shock numbers (*p* < 0.001 MK-801 vs. MK-801 + PA-Glu 0.1) (Figure 8A). In all MK-801 + MS-249 groups, animals received significantly more shocks than control animals (*p* < 0.01 CDX vs. MK-801 + MS-249 0.01 mg/kg, *p* < 0.001 CDX vs. MK-801 + MS-249 0.1 mg/kg, *p* < 0.05 CDX vs. MK-801 + MS-249 1 mg/kg) (Figure 8B). A substantial increase in the number of shocks, with a less pronounced increase in the number of entrances in MK-801 + MS-249 groups, illustrates the lack of escape reaction after shock is delivered.

**Figure 9:**
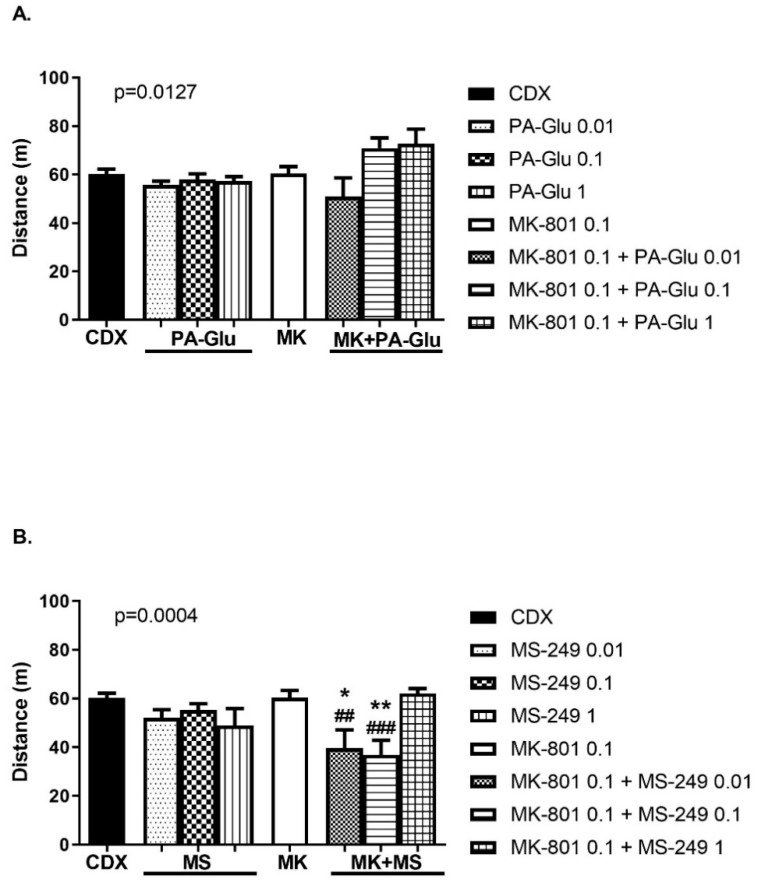
Distance walked in CM. (**A**) Post hoc test did not reveal any significant changes in distance walked between groups. (**B**) MK-8101 + MS-249 application in lower doses significantly decreased the distance walked compared to both vehicle and MK-801 groups. Data were analyzed by 1-way ANOVA followed by Dunnett’s post hoc test. * *p* <0.05, ** *p* <0.01 vs. CDX controls, ^##^ <0.01, ^###^ <0.001 vs. MK-801-treated rats.

The type of neuroactive steroid treatment significantly influenced locomotor activity. ANOVA analyzes for both neuroactive steroids showed a significant influence of treatment on distance walked, F(7, 60) = 2.838, *p* = 0.0127 for PA-Glu (Figure 9A) and F(7, 54) = 4.704, *p* = 0.0004 for MS-249 administration (Figure 9B). However, the activity of animals treated with PA-Glu of all doses alone or in combination with MK-801 remained unchanged compared to controls or MK-801-treated animals (Figure 9A). MK-801 + MS-249 co-administration led to decreased locomotion in MS-249 doses of 0.01 (*p* < 0.01) and 0.1 mg/kg (*p* < 0.001) compared to the MK-801 group and in MK-801 + MS-249 0.01 mg/kg (*p* < 0.05) and MK-801 + MS-249 0.1 mg/kg (*p* < 0.01) dose compared to CDX group (Figure 9B).

## 4. Discussion

The present study aimed to compare the antipsychotic effects of negative NMDAR modulator PA-Glu to positive NMDAR modulator MS-249 in a battery of behavioral tests (OF, EPM, PPI, PA, CM). In addition, we evaluated electrophysiological recordings in order to compare their effects on NMDAR currents to endogenous neurosteroids, PA-S and PE-S. Schizophrenia is characterized by NMDAR hypofunction; thus, boosting the receptor function should positively affect performance in the MK-801-induced schizophrenia model. To pursue this line of inquiry, animals were administered neuroactive steroids alone or in combination with MK-801 that irreversibly inhibits the responses of NMDARs (Figure 2A).

In the open field, neither PA-Glu nor MS-249 administration affected locomotor activity (Figure 3). We chose the MK-801 dose that is known to alter cognitive performance while the locomotor activity remains largely intact. However, co-application of MK-801 with MS-249 at lower doses (0.1 and 1 mg/kg) significantly enhanced locomotion (Figure 3B). This potentiating effect on activity was not observed in MK-801 + PA-Glu treated rats (Figure 3A) or in the highest dose of MS-249 applied with MK-801 (Figure 3B). PA-Glu had no adverse effects on locomotion despite its negative modulation of NMDARs as NMDAR antagonists are well-known for their hyperlocomotion-inducing effects [48,49,50].

The anxiety assessment in the elevated plus maze showed an anxiolytic effect of PA-Glu in both parameters—the time spent in open arms (Figure 4A) and latency to enter open arms (Figure S1A). The most robust effect was observed at the highest dose of 10 mg/kg. However, latency was significantly reduced in all PA-Glu-treated animals when compared to controls. The locomotor activity was significantly increased in rats administered PA-Glu 10 mg/kg. Still, it is unlikely that the increased time in open arms could be attributable solely to increased locomotion (Figure S2A). As seen in OF, none of the doses of PA-Glu applied altered locomotion. It is more plausible that exposure to EPM resulted in anxiety-related suppression of locomotion in the control group. Indeed, our control animals showed enhanced anxiety measured by open arm time compared to reports from literature (3% vs. 20% of the time in open arms usually reported [51,52]). Contrary to PA-Glu, MS-249 did not exert anxiolytic properties (Figure 4B and Figure S1B).

Three cognitive tests were carried out to evaluate sensorimotor gating, learning and memory—pre-pulse inhibition of startle response, passive avoidance and carousel maze. PPI measures a neurological process of filtering out irrelevant sensory information during the early stages of processing so that attention may be focused on more salient features of the environment [53]. PPI deficits are frequently reported among schizophrenia patients [54] and in animal models of schizophrenia [55]. The PA step-through test does not require active task solving, and mere inactivity/passivity results in a successful outcome. In contrast to PA, CM is a more demanding test requiring active avoidance. Thus, it is more aversive and stressful.

The PPI of acoustic startle response is not considered a classical cognitive test per se; however, there is some evidence that PPI positively correlates with performance on specific neurocognitive tasks [56]. MK-801 administration resulted in PPI deficits, a finding consistent with the literature [57,58]. PA-Glu dose-dependently decreased PPI compared to controls, with the 10 mg/kg dose reaching the level of significance (Figure 5A). MS-249 did not show any negative effect when applied alone (Figure 5B). Neither neuroactive steroid reversed PPI deficits caused by MK-801 co-administration. Its inhibitory action at NMDARs might explain the detrimental impact of PA-Glu on PPI. Both noncompetitive and competitive NMDAR antagonists disrupt PPI consistently across several laboratories, conditions, strains and animal suppliers [59].

The latency to cross from the illuminated to the dark compartment in PA was significantly reduced in the MK-801 group, indicating impaired learning and memory. PA-Glu dose-dependently shortened the latency; in the highest dose group (10 mg/kg), the latency was comparable to the MK-801 group (Figure 6A). The performance of MS-249-treated animals in PA was unaffected; no memory deficit was observed compared to the control group (Figure 6B). The administration of both PA-Glu and MS-249 in low and medium doses reversed the memory deficit elicited by MK-801 co-administration. The highest doses of both neuroactive steroids failed to restore cognitive functioning in MK-801-treated rats.

A single administration of either neuroactive steroid did not affect performance in the carousel maze compared to vehicle-treated controls. MK-801 application induced cognitive impairment; the number of entrances and shocks was significantly increased while locomotion remained unchanged. PA-Glu at the dose 0.1 mg/kg normalized cognitive impairment caused by MK-801 co-application (Figure 7A and Figure 8A). None of the MS-249 doses had a therapeutic effect when administered with MK-801 (Figure 7B and Figure 8B).

The increased number of shocks an animal receives reflects the lack of escape reaction. If the animal fails to escape from the to-be-avoided sector, it might get up to 5 shocks upon single sector entrance. This passive behavior is indicated by reduced locomotor activity and might be a consequence of stress-induced anxiety. Combining two lower doses of MS-249 with MK-801 resulted in a significantly increased number of shocks (Figure 8B) and decreased locomotor activity (Figure 9B). The magnitude of increase in the number of shocks was not seen in the number of entrances, indicating an impaired escape reaction—rats received more shocks per single entrance indicating passive behavior. Moreover, CM, contrary to PA, requires an active approach for successful performance. MS-249 + MK-801 application resulting in passive behavior would manifest as memory deficit in the CM test but as a memory-enhancing effect in the PA test.

The failure of MS-249 to prevent MK-801-induced behavioral impairment in CM might be partly explained by its effect at NMDARs. The results of our electrophysiological experiments show that MS-249 was more effective in the potentiation of NMDAR-induced currents in comparison to endogenous PE-S (Figure 2C,D). On the other hand, the inhibitory effect of PA-Glu at NMDARs was comparable to endogenous PA-S (Figure 2B,D). In addition, PA-Glu demonstrated an allosteric modulatory effect on GABA_A_Rs at 1 µM concentration measured by [^35^S]TBPS displacement while MS-249 did not affect GABA_A_Rs. The concentration–response curve measured (10 nM–100 µM) for [^35^S]TBPS inhibition revealed PA-Glu IC_50_ value of 7.5 µM. These results are in agreement with data measured for PA-Glu on pyramidal neurons of the rat hippocampus using the patch-clamp technique in the study of Bukanova et al. The enhancing effect of PA-Glu on GABA_A_Rs was reported at 5–50 µM concentrations with an EC_50_ value of 7 ± 3 μM [60]. These results suggest that the behavioral effect of PA-Glu is the consequence of both NMDARs and GABA_A_Rs modulation. Conversely, MS-249 does not affect GABA_A_Rs, and the modulatory effect on NMDARs is significantly greater than the effect of PE-S naturally occurring in the brain. Moreover, some behavioral effects of PE-S are thought to be exerted through its conversion to pregnenolone [61] acting as a precursor for all brain-derived neuroactive steroids. Pregnenolone sulfate increases allopregnanolone brain levels [28] and allopregnanolone acts as a positive modulator of GABA_A_Rs with anxiolytic properties [18]. It was documented that MK-801-induced memory deficits in the PA test were counteracted by pregnenolone sulfate administration due to its ability to increase brain accumulation of allopregnanolone [62].

NMDAR antagonists and GABA_A_R agonists display potent anxiolytic properties, suggesting both neurotransmitter systems’ involvement in the regulation of anxiety. This was shown by PA-Glu treated rats in EPM. In behavioral tests, the use of aversive stimuli, such as footshocks, might lead to anxiety in tested animals. The exposure to acute stress potentiated the anxiety response [63], suggesting that the neurocircuitry governing the stress response and anxiety overlap. Acute stress increases extracellular glutamate levels in the amygdala [64], prefrontal and frontal cortex [65,66] leading to stimulation of glutamate receptors, including NMDARs. Acute mild stress might facilitate working memory [65]; however, the stress severity and the complexity of the task might hamper the positive effect of stress on memory [67] and cause anxiety instead. A combination of elevated glutamate levels coupled with positive NMDARs modulation might excessively enhance glutamate transmission. Therefore, MS-249 injection might worsen performance in vulnerable individuals under highly stressful conditions in animals with normal NMDAR function. MS-249 1 mg/kg application altered the escape reaction in CM; three animals received more than 70 shocks, and one walked less than 20 m. The performance of the MS-249 group did not significantly differ from neither the control nor the MK-801 group (Figure 7B and Figure 8B). On the other hand, GABAergic transmission is rapidly decreased following acute stress [68]. Neurosteroids enhancing GABA_A_ receptor function may minimize the reduction in GABAergic inhibitory transmission and restore HPA axis homeostatic control [69].

The more profound impairment of MK-801 + MS-249-treated animals compared to the MK-801-treated group in CM might be explained by the preferential blockade of NMDARs by MK-801 residing on GABAergic interneurons, which are 10-fold more sensitive to NMDAR antagonists than the pyramidal neurons [70]. MK-801-induced reduction in the activity of GABA neurons precedes increased excitation of pyramidal neurons in mPFC [71]. The inhibition of GABA neuron activity by MK-801 coupled with stimulation of pyramidal neurons by MS-249 and by stress-released glutamate worsened the performance in MK-801 + MS-249 animals. Thus, MS-249 was able to ameliorate performance in the schizophrenia model in the PA test, but failed to do so in the CM test, characterized by increased stress exposure. A complementary explanation of this phenomenon might be that positive NMDAR modulation leading to, e.g., prolonged channel-open time and increased frequency or probability of channel opening, facilitates MK-801 binding to NMDAR. Although, the level of stress likely plays a role to some extent, since in non-stressful tests such as OF, MK-801 + MS-249 application resulted in increased locomotion (Figure 3B). However, in the CM test, where rats received footshocks, rats administered MK-801 + MS-249 showed suppressed locomotion (Figure 9B). The reason why the highest dose of MS-249 did not have such a detrimental effect on behavior in MK-801-treated rats as lower doses remains to be elucidated.

The effect of GABA_A_R and NMDAR modulation reaches beyond mere stress and anxiety modulation. NMDARs and GABA_A_Rs contribute to the excitatory–inhibitory brain circuit balance, and distortion of the finely tuned interplay between excitation and inhibition may manifest as schizophrenia and autism spectrum disorders, among others. It is plausible that a net change in network excitability determines the overall effect of neurosteroid administration. The actual extracellular concentration of neurosteroid and glutamate in the brain and basal NMDAR activity might be decisive factors determining the outcome of behavioral tests. Furthermore, neurosteroids are known to exhibit pleiotropic actions mediated through the number of receptor systems, (GABA_A_Rs, NMDARs, AMPA receptors, σ1 receptors, nicotine receptors) [72,73,74]; therefore, we cannot rule out the possibility that the behavioral effect of our neuroactive steroids is not mediated solely via NMDARs or GABA_A_Rs. In addition, the neuroactive steroids might be further broken down into active metabolites with their own receptor-modulatory activity, as is the case of PE-S.

## 5. Conclusions

Our results with MS-249 show that enhancement of NMDAR function might result in non-specific behavioral responses. Positive NMDAR modulation can influence other neurobiological processes besides memory formation, e.g., anxiety and response to stress, confounding desirable treatment outcomes with memory enhancers acting via NMDARs. Moreover, neurosteroids are not selective NMDAR modulators, and consequently, their overall behavioral effect is a combination of various receptor-modulatory actions.

## Data Availability

Data presented in this study are available at Mendeley Data at http://dx.doi.org/10.17632/5bg2z4hrdw.2.

## Supplementary Materials

The following are available online at https://www.mdpi.com/article/10.3390/biom11071026/s1, Figure S1: Latency to enter open arms in EPM. (A) Latency to enter open arms analyzed by 1-way ANOVA showed a significant reduction in all PA-Glu doses administered. (B) MS-249 had no effect on anxiety. Dunnett’s post-hoc test revealed these group differences: * <0.05, ** <0.01 vs. CDX controls, Figure S2: Distance in EPM. (A) Highest dose of PA-Glu significantly increased distance walked while MS-249 showed no effect on loco-motion in EPM, (B) Data were analyzed by 1-way ANOVA followed by Dunnett’s post-hoc test. *** <0.001 vs. CDX controls. Table S1: Summary of the results. The table depicts mean group values obtained from all behavioral experiments with both neuroactive stroids. The number of animals per group is provided as well. Data were analased using 1-way ANOVA followed by Dunnett’s post-hoc test when appropriate. MK-801 0.1 mg/kg was applied in all experiments but PPI where the dose 0.3 mg/kg was used. * <0.05, ** <0.01, *** <0.001 vs. CDX and # <0.05, ## <0.01, ### < 0.001 vs. MK-801.
