# Supplementary material for: Pitfalls of NMDA Receptor Modulation by Neuroactive Steroids. The Effect of Positive and Negative Modulation of NMDA Receptors in an Animal Model of Schizophrenia
Source: Biomolecules. 2021 Jul 14;11(7):1026. doi: 10.3390/biom11071026 (PMC8301783; doi:10.3390/biom11071026)
Supplement: Supplementary file 1 [file biomolecules-11-01026-s001.zip › biomolecules-1262596-supplementary.pdf]

biomolecules

Supplementary

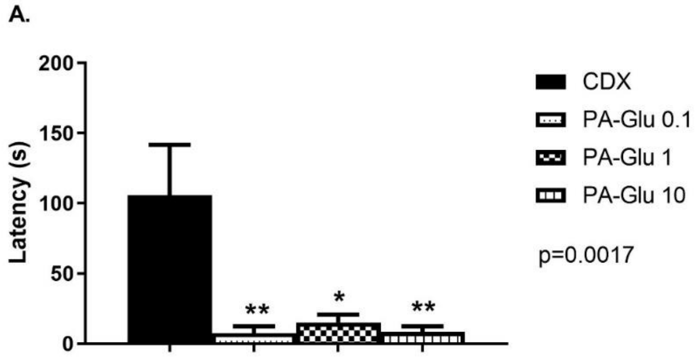

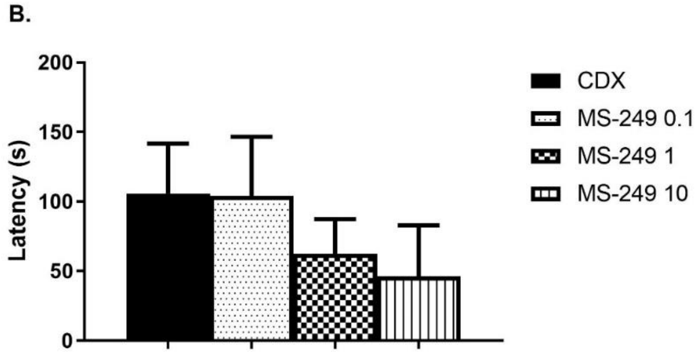

Figure S1. Latency to enter open arms in EPM. A. Latency to enter open arms analyzed by 1-way ANOVA showed a significant reduction in all PA-Glu doses administered. **B.** MS-249 had no effect on anxiety. Dunnett´s post-hoc test revealed these group differences: *<0.05, **<0.01 vs CDX controls.

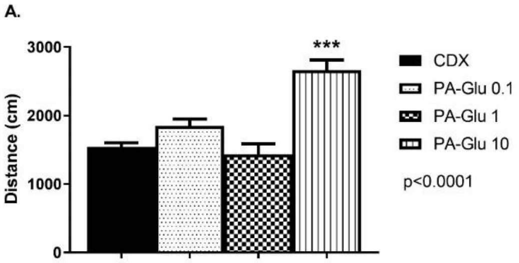

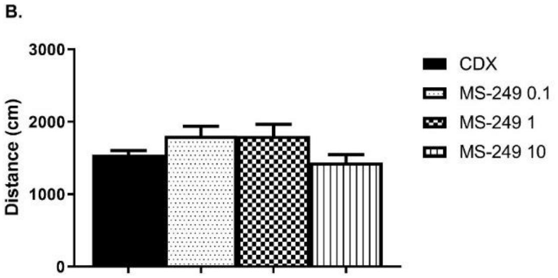

Figure S2. Distance in EPM. A. Highest dose of PA-Glu significantly increased distance walked while MS-249 showed no effect on locomotion in EPM, **B.** Data were analyzed by 1-way ANOVA followed by Dunnett´s post-hoc test. ***<0.001 vs CDX controls.

Table S1. Summary of the results. The table depicts mean group values obtained from all behavioral experiments with both neuroactive stroids. The number of animals per group is provided as well. Data were analased using 1-way ANOVA followed by Dunnett´s post-hoc test when appropriate. MK-801 0.1 mg/kg was applied in all experiments but PPI where the dose 0.3 mg/kg was used. *<0.05, **<0.01, ***<0.001 vs CDX and #<0.05, ##<0.01, ###< 0.001 vs MK-801.

drug	dose	OF		EPM				PPI		PA		CM			
	(mg/kg)	distance	n	TOA	LAT OA	distance	n	% of PPI	n	latency	n	entrances	shocks	distance	n
CDX		71.13	8	8.82	105.83	1542.67	10	47.53	12	214.91	12	8.87	16.25	60.30	8
MK-801	0.1/0.3	101.6	8	N/A				-33.10***	5	17.21***	8	23.81***	77.06**	60.37	16
PA-Glu	0.01	N/A						N/A		N/A		9.2##	20.4##	55.73	5
	0.1	94.2	6	21.02	7.53**	1849.77	9	47.90###	5	222.29###	7	6.33###	10.67###	57.97	6
	1	78.37	6	14.61	15.04*	1434.90	8	31.17###	8	137.37##	6	10.43##	17.71##	57.38	7
	10	84.13	6	98.44***	8.41**	2662.61***	8	4.31**	8	47.84*	5	N/A			
PA-Glu + MK-801	0.01	N/A		N/A				N/A		N/A		17.6	73.4	50.90	5
	0.1	104.33	6					-25.63***	4	138.173#	7	7.88###	16.38###	70.90	8
	1	76.50	5					-12.38***	8	135.613#	8	21.58*	62.58	72.80	12
	10	102.15	8					-33.98***	8	28.98***	6	N/A			
MS-249	0.01	N/A						N/A		N/A		7.4##	17.00##	52.04	5
	0.1	102.43	6	10.72	104.08	1809.57	9	38.47###	5	235.56###	8	6.00###	14.33###	55.27	6
	1	87.11	7	13.17	62.64	1804.16	7	42.24###	6	195.84###	7	16.71	59.43	48.98	7
	10	94.93	7	13.19	46.25	1438.54	8	44.57###	8	267.8###	8	N/A			
MS-249 + MK-801	0.01	N/A		N/A				N/A		N/A		23.33**	104.00**	39.63*##	6
	0.1	118.53**	6					-41.04***	5	134.90	8	27.83***	122.00***	36.86**###	6
	1	122.10**	6					-25.18***	5	165.19##	15	21.88*	72.63*	62.12	8
	10	93.02	6					10.49	6	54.10***	7	N/A			
